# Metabolomics analysis reveals the accumulation patterns of flavonoids and phenolic acids in quinoa (*Chenopodium quinoa* Willd.) grains of different colors

**DOI:** 10.1016/j.fochx.2023.100594

**Published:** 2023-02-06

**Authors:** Guangtao Qian, Xiangyu Li, Heng Zhang, Hailong Zhang, Jingwen Zhou, Xiaohui Ma, Wei Sun, Wei Yang, Ruikun He, Atia-tul Wahab, Huihua Wan, Lixin Li

**Affiliations:** a Key Laboratory of Saline-alkali Vegetation Ecology Restoration, Ministry of Education, College of Life Sciences, Northeast Forestry University, Harbin 150040, China; b Institute of Crop Resources, Heilongjiang Academy of Agricultural Sciences, Harbin 150086, China; c Key Laboratory of Beijing for Identification and Safety Evaluation of Chinese Medicine, Institute of Chinese Materia Medica, China Academy of Chinese Medical Sciences, Beijing 100700, China; d State Key Laboratory of Plant Molecular Genetics, Shanghai Center for Plant Stress Biology, Center for Excellence in Molecular Plant Sciences, Chinese Academy of Sciences, Shanghai 201602, China; e Center for Molecular Medicine and Drug Research, International Center for Chemical and Biological Sciences, University of Karachi, Karachi 75270, Pakistan; f Byhealth Institute of Nutrition & Health, Guangzhou 510663, China

**Keywords:** Quinoa grains, UPLC-ESI-MS/MS, Metabolic profiles, Flavonoids, Phenolic acids

## Abstract

Quinoa grains are gaining increasing popularity owing to their high nutritional merits. However, only limited information is available on the metabolic profiles of quinoa grains. In this study, we determined the metabolic profiles of black, red, and white quinoa grains via an ultraperformance liquid chromatography–electrospray ionization–tandem mass spectrometry (UPLC–ESI–MS/MS)-based metabolomics. A total of 689 metabolites were identified, among which 251, 182, and 317 metabolites displayed different accumulation patterns in the three comparison groups (Black vs Red, Black vs White, and Red vs White), respectively. In particular, flavonoid and phenolic acid contents displayed considerable differences, with 22 flavonoids, 5 phenolic acids, and 1 betacyanin being differentially accumulated among the three quinoa cultivars. Additionally, correlation analysis showed that flavonoids and phenolic acids could act as betanin co-pigments in quinoa grains. In conclusion, this study provides comprehensive insights into the adequate utilization and development of novel quinoa-based functional foods.

## Highlights

• A total of 689 metabolites were identified in the grains of three quinoa varieties.
• 102 flavonoids and 97 phenolic acids were identified in quinoa grains.
• Most differential metabolites were significantly more abundant in colored quinoa grains than in white grains.
• Flavonoids and phenolic acids could act as co-pigments of betanin in quinoa grains.

## 1 Introduction

Quinoa (*Chenopodium quinoa* Willd.), an annual pseudocereal crop of the Amaranthaceae family, is native to the Andean region in South America. Quinoa grains are naturally gluten- and cholesterol-free, and have high protein content and excellent amino acid composition (Filho et al., 2017). In fact, these grains contain all essential amino acids required in the human body along with several natural antioxidant elements, vitamins, dietary fiber, and minerals (Dakhili, Abdolalizadeh, Hosseini, Shojaee-Aliabadi & Mirmoghtadaie, 2019). In addition, high-risk populations, especially people with gluten allergy, diabetes, anemia, or obesity, may benefit from the intake of these grains that have low starch content (Li et al., 2018, Zevallos et al., 2014). The Food and Agriculture Organization of the United Nations (FAO) declared 2013 as the international year of quinoa to support its acceptance as a “functional food” (https://www.fao.org/quinoa-2013/en/).

Quinoa grains can be classified as white, red, or black according to their pigmentation (Tang et al., 2015). Phenolic compounds, including phenolic acids, flavonoids, and tannins are one of the most important secondary metabolites in quinoa (Al-Qabba et al., 2020). The total phenolic concentration in quinoa grains is reported to be 1.67–3.08 g/kg DW (Han et al., 2019). Furthermore, phenolics in quinoa grains have been demonstrated to possess excellent antioxidant activities (Chandrasekara and Shahidi, 2011, Tang et al., 2016). To date, approximately 37 flavonoids and 29 phenolic acids have been identified and isolated from quinoa seeds, flour, leaves, and sprouts (Lin et al., 2019). The distribution, content, and antioxidant activities of phenolic compounds significantly differ among various quinoa varieties, tissues and developmental stages. For example, sprouts contain higher levels of kaempferol and quercetin glycosides than grains, whereas bran contains a high rutin concentration. Moreover, roots and sprouts possess higher antioxidant activities than other tissues (Lin et al., 2019, Morales et al., 2020). Han et al. (2019) found that darker quinoa had a higher content of phenolic compounds as well as a stronger antioxidant activity than lighter varieties. The predominant flavonoids in quinoa grains include rutin, kaempferol, and quercetin (Ng & Wang, 2021). Furthermore, some varieties contain naringin, myricetin, eriodictyol, and isorhamnetin, which possess different pharmacological activities, such as reducing the incidence of cardiovascular and neurodegenerative diseases (Zhang et al., 2020). Phenolic acids, such as vanillic acid, gallic acid, ferulic acid, p-hydroxybenzoic acid, and their derivatives were observed in differently colored quinoa genotypes. Their levels varied significantly among quinoa grains of different colors (Han et al., 2019, Rocchetti et al., 2019).

Metabolomics is an effective methodology for analyzing the dynamics of metabolites exposed to endogenous or exogenous factors or stimuli (Chen et al., 2013). Previous researchers have used high-performance liquid chromatography (HPLC) coupled with UV, HPLC-ESI-MS/MS, or even UHPLC-QTOF-MS/MS to analyze the components of different quinoa varieties (Gomez-Caravaca et al., 2011, Rocchetti et al., 2019). However, studies have rarely been carried out on large-scale detection, identification, and quantification of all chemical components and nutrients in quinoa grains. Widely targeted metabolomics can determine the effects of genetic, physiological, and ecological factors on the secondary metabolites of various crops. For example, the phenotypes of different varieties of wheat and tartary buckwheat are closely associated with their metabolites (Wang et al., 2020, Yang et al., 2020). Therefore, a widely targeted metabolomic approach based on UPLC–ESI–MS/MS was employed to identify and quantify the primary and secondary metabolites in differently colored quinoa grains. Thereafter, three quinoa cultivars were analyzed to determine the correlation between the cultivars and their metabolites. Finally, functional components associated with health benefits (flavonoids and phenolic acids) were compared among these cultivars. This study provides valuable information and critical guidance for the development of quinoa-based functional foods.

## 2 Materials and methods

### 2.1 Reagents and chemicals

LC–MS grade acetonitrile, formic acid, and methanol were obtained from Honeywell (Morris, NJ, USA). Pure distilled water was acquired from a Milli-Q 50 system (Millipore Iberica SA, Madrid, Spain).

### 2.2 Sample preparation and extraction

**Fig. 1:**
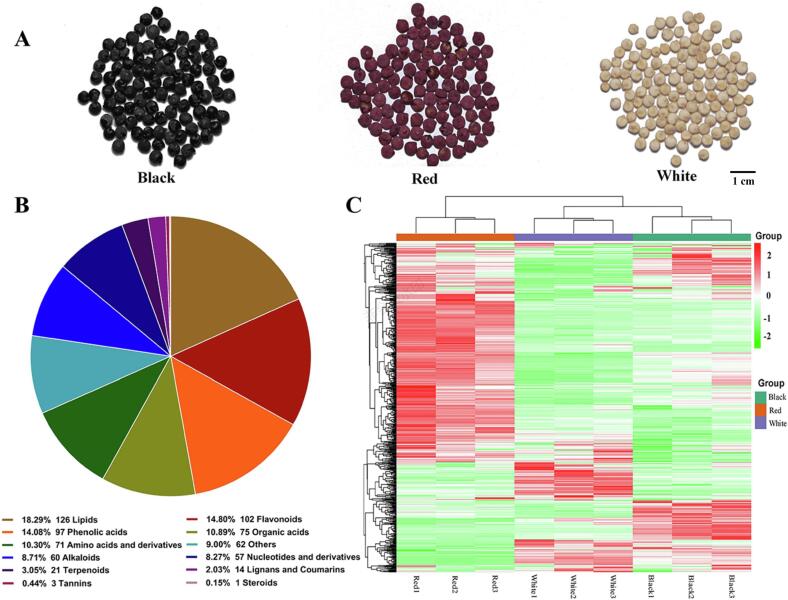
Heatmap analysis and identified metabolites in quinoa grains. A: Photographs showing black, red, and white quinoa grains. B: Component analysis of the identified metabolites. Type, quantity, and proportion of all identified metabolites are presented below the graph. C: HCA analyses of metabolites identified in three quinoa cultivars. Metabolite values are denoted by a unique color. Red represents high levels and green represents low levels; the color key is displayed on the right of the heatmap. (For interpretation of the references to colour in this figure legend, the reader is referred to the web version of this article.).

Three representative quinoa cultivars with different grain colors, including “Yana” (black quinoa grains from Bolivia, Black), “Pasan Ralle” (red quinoa grains from Bolivia, Red), and “QQ87” (white quinoa grains from Argentina, White) were selected for this study (Fig. 1A). All cultivars of quinoa seeds were sown in Wulan, Qinghai, China (36°93′N; 98°48′E) with the same cultivation environment in May 2020. The quinoas were grown in an open field with soil conditions using the randomized complete block design. Each cultivar was sown in an isolated manner to avoid crossbreeding. The standard field management was carried out during the plants growth. Mature grains without damage or mildew were randomly harvested from plants in October 2020. After drying, these quinoa grains were stored at the National Gene Bank of Traditional Chinese Medicine before metabolomics.

Grains of each quinoa variety were vacuum freeze-dried for 24 h. Thereafter, the grains were ground in a mixer mill (MM 400, Retsch, Haan, Germany) at 30 Hz for 120 s. Subsequently, 100 mg of each powder was weighed, extracted using 1.0 mL of 70 % aqueous methanol, stored at 4 ℃ overnight, vortexed for 5 min (1 min each time), and centrifuged at 13,800*g* for 10 min. The supernatants were finally filtered with a 0.22 μm millipore filter. Each sample was replicated three times using three individual plants (Black 1, Black 2, Black 3; Red 1, Red 2, Red 3; White 1, White 2, White 3). Differently colored quinoa grains were mixed to generate the quality control (QC) samples. A QC sample and every-three test samples was injected during the analysis to verify stability and accuracy.

### 2.3 UPLC conditions and ESI-Q TRAP-MS/MS

An UPLC–ESI–MS/MS system (UHPLC, SHIMADZU Nexera X2 system, Kyoto, Japan; MS, Applied Biosystems 4500 Q TRAP, Foster City, CA, USA) was employed to analyze the grain sample extracts. The following operating parameters and specifications were employed as previously reported (Wang et al., 2020): column temperature, 40 °C; column, Agilent SB-C18 (pore size 1.8 µm, length 100 mm × 2.1 mm); mobile phase, eluent A (0.1 % formic acid), and eluent B (acetonitrile containing 0.1 % formic acid); sample injection volume, 2 μL; flow rate, 0.3 mL·min^−1^. The following gradient program was employed for the analysis: starting conditions of 5 % B; 5–95 % B, 9 min; 95 % B for 1 min; 95–5 % B, 11.1 min; and 5 % B for 2.9 min. To decrease the analysis bias, samples were injected in a fully randomized sequence. The effluent was transported to an ESI-Q-TRAP-MS system.

Linear ion trap (LIT) and triple quadrupole (QQQ) scans were acquired on a 4500 Q TRAP LC/MS/MS system, coupled with an ESI Turbo Ionspray interface, operated in both positive and negative ionization modes and processed by Analyst 1.6 software (AB Sciex). Mass spectrometry analysis and ESI source conditions were as previously described (Chu et al., 2019): ion source, turbo spray; source temperature, 500 °C; ion spray voltage (IS), +5.5/−4.5 KV; pressures of gas I (GSI), gas II (GSII), and curtain gas (CUR) were set at 345, 414, and 172 kPa, respectively, and collision gas (CAD), high. QQQ scans were obtained as MRM experiments, with the collision gas (nitrogen) set to 34 kPa. Declustering potential (DP) and collision energy (CE) for individual MRM transitions were performed with further DP and CE optimization.

### 2.4 Qualitative and quantitative analyses of metabolites

The stepwise multiple ion monitoring-enhanced product ions (MIM-EPI) technique was used to collect and process MS data. For the qualitative analysis of metabolites in quinoa grains, the primary and secondary MS data were used to annotate metabolites based on the selfbuilt metware database (MWDB) (Chen et al., 2013, Cao et al., 2022) and the public metabolite database (MassBank, HMDB, ChemBank, PubChem, and METLIN). Information regarding fragmentation ions with the desired characteristics was obtained via QQQ after excluding the initial interference from non-target ions. After obtaining the basic MS data of the metabolites, the relative content of each metabolite in the different samples was represented as chromatographic peak areas; MS spectrometry data were integrated and corrected using MultiaQuant software.

### 2.5 Statistical analysis

Filtered and processed data were submitted to R software (https://www.r-project.org/) for principal component analysis (PCA), orthogonal projections to latent structure-discriminant analysis (OPLS-DA), and hierarchical clustering analysis (HCA) as previously described (Wang et al., 2016). OPLS-DA is an efficient supervised method for screening potential markers with low correlations among groups (Boccard & Rutledge, 2013). After screening for the orthogonal variables for all metabolites, the OPLS-DA model was validated using 200 random permutations (Trygg & Wold, 2002). To screen out the differentially accumulated metabolites (DAMs) between two cultivars, variables in project importance (VIP) ≥ 1 and fold change ≥ 2 or ≤ 0.5 were set as the selection criteria. The Kyoto Encyclopedia of Genes and Genomes (KEGG) database was used to annotate and classify the DAM functions between the differently colored quinoa grains. GraphPad Prism 8 was used for graphic drawing. Duncan’s multiple range test was used to determine statistical significance with a *P* value <0.05; data are expressed as mean ± SD.

## 3 Results and discussion

### 3.1 Metabolite profiling analysis of quinoa grains

UPLC–ESI–MS/MS-based widely targeted metabolomics approach was applied to determine the associated metabolites and DAM composition in the grains of three quinoa cultivars. The total ion current (TIC) analysis of the QC samples was used to examine the consistency of metabolite extraction and detection. TIC curves overlapped with the results of the metabolite detection (Fig. S1A, S1B). Retention times and peak intensities remained constant when an identical sample was identified at a different time, which indicated the stability of the signal. The repeatability and reliability of our metabolomic data were markedly enhanced by instrumental stability. Furthermore, Pearson’s correlation coefficient was determined among the replicated intragroup samples, which ensured good homogeneity (R^2^ close to 1, Fig. S1C).

Following quality evaluation, a total of 689 metabolites were tentatively identified including 391 primary metabolites (lipids, amino acids and derivatives, nucleotides and derivatives, organic acids, and others) and 298 secondary metabolites (flavonoids, phenolic acids, alkaloids, terpenoids, lignans and coumarins, tannins, and steroids) (Table S1). Lipids (126, 18.3 %), flavonoids (102, 14.8 %), phenolic acids (97, 14.1 %), organic acids (75, 10.9 %), and amino acids and derivatives (71, 10.3 %) accounted for a substantial proportion of all metabolites (Fig. 1B). Nine samples could be clearly classified into three groups based on the HCA heatmap (Fig. 1C). Furthermore, the relative content of metabolites in red grains markedly differed from that of white and black grains, thereby indicating that the composition of quinoa grains differs significantly among the three cultivars. Accordingly, the metabolite profiles of different quinoa cultivars were strongly influenced by genetic variation.

In the present study, a total of 102 flavonoids (2 chalcones, 7 dihydroflavones, 3 dihydroflavonols, 38 flavones, 46 flavonols, and 6 flavonoid carbonosides) were identified. We found that orientin, diosmetin, rutin, quercetin, prunin, kaempferol, cynaroside, linarin, aromadendrin, isorhamnetin, and kaempferitrin were modified by various glycosidic bonds in differently colored quinoa grains, and were primarily glycosylated at positions 8-C, 6-C, 3-O, and 7-O. Among these, kaempferol, isorhamnetin, diosmetin, aromadendrin, and quercetin were glycosylated at positions 3-O and 7-O by neohesperidoside, glucoside, or glucuronide. Six flavonoids (luteolin-7-*O*-glucoside, luteolin-7-*O*-rutinoside, quercetin-3-*O*-neohesperidoside, quercetin-3-*O*-rutinoside-7-*O*-rhamnoside, quercetin-3-*O*-(2″-*O*-arabinosyl)-rutinoside, and kaempferol-3-*O*-rutinoside-7-*O*-rhamnoside) were identified as the most abundant in the three quinoa cultivars. The content of quercetin-3-*O*-neohesperidoside, quercetin-3-*O*-rutinoside-7-*O*-rhamnoside and quercetin-3-*O*-(2″-*O*-arabinosyl)-rutinoside in white grains was 1.03–7.23 times higher than that in black and red grains. Likewise, the content of luteolin-7-*O*-glucoside, luteolin-7-*O*-rutinoside and kaempferol-3-O-rutinoside-7-O-rhamnoside was higher in red quinoa grains than that in black and white grains.

Additionally, quinoa grains were found to contain 97 phenolic acids with different levels. According to previous studies, the phenolic acids associated with quinoa grains also exist as aglycones and glycosylated derivatives (Hussain et al., 2021). In this study, ferulic acid, vanillic acid, sinapic acid, *p*-coumaric acid, protocatechuic acid, caffeic acid, and salicylic acid occurred both as aglycones and glycosylated derivatives. However, benzoic acid, syringic acid, and gentisic acid existed only as glycosylated derivatives, whereas homogentisic acid existed only as aglycon.

### 3.2 PCA and OPLS-DA of the three quinoa cultivars with different colors

**Fig. 2:**
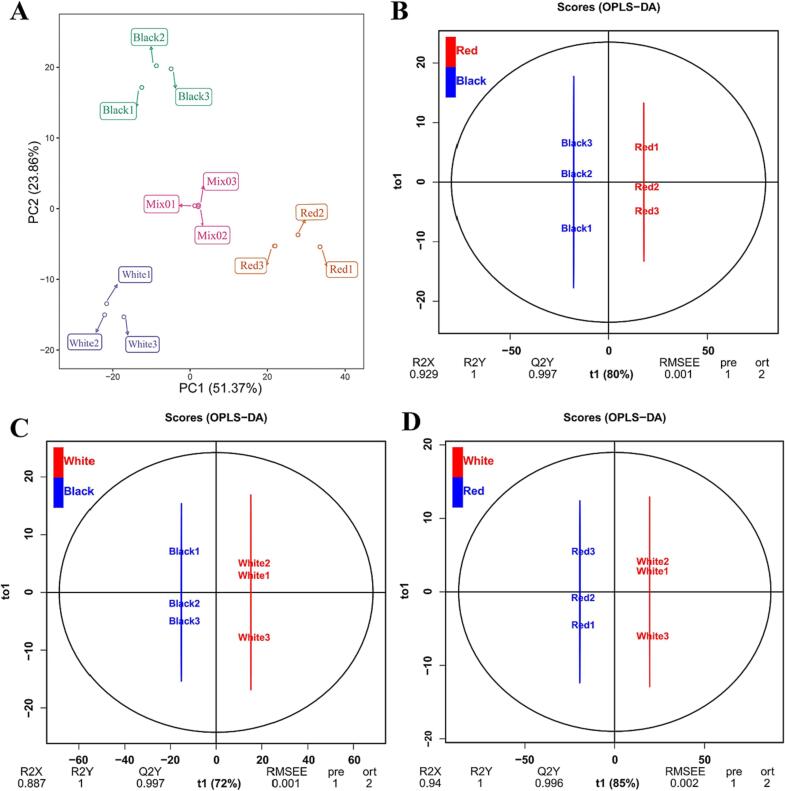
PCA and OPLS-DA of the three quinoa cultivars. A: PCA score plot of the metabolite profiles of Black, Red, White, and Mixed; different colors represent different experimental groups: green = black; orange = red; purple = white; and pink = QC sample; the abscissa and ordinate indicate the first and second principal component (PC1 and PC2), respectively. Score plots for PC1 and PC2 show high cohesion within groups and good separation among the Black, White, and Red samples. B, C and D: Score plots of the OPLS-DA model for Black vs Red, Black vs White, and Red vs White, respectively. (For interpretation of the references to color in this figure legend, the reader is referred to the web version of this article.).

PCA is beneficial in analyzing the internal structure of numerous variables using only a few principal components (Clark et al., 2019). In the PCA score plot, the cumulative contribution rate of two principal components (PC1 51.37 % × PC2 23.86 %) reached 75.23 %. As shown in Fig. 2A, Black, Red, and White could readily be separated, which suggested that metabolites of the three quinoa cultivars were obviously different; the three biological repeats of each cultivar formed a compact cluster. The results of these experiments indicated that the materials had sufficient reproducibility and were suitable for subsequent qualitative and quantitative analyses.

OPLS-DA models were implemented to compare the DAMs among different quinoa cultivars. High predictability (Q^2^) is a crucial parameter that represents the predictive capacity of the model. When Q^2^ is >0.9 and the goodness of fit is strong (R^2^X, R^2^Y approximately 1), the model is considered excellent and stable. Herein, we obtained high Q^2^, R^2^X, and R^2^Y values to assess the validity of the OPLS-DA model among the samples. In this study, all metabolites of quinoa grains were evaluated using pairwise comparison based on the OPLS-DA model to determine the difference between Black and Red (Q^2^ = 0.962, R^2^X = 0.671, and R^2^Y = 0.980; Fig. 2B), Black and White (Q^2^ = 0.934, R^2^X = 0.6, and R^2^Y = 0.996; Fig. 2C), and Red and White (Q^2^ = 0.974, R^2^X = 0.743, and R^2^Y = 0.986; Fig. 2D). A Q^2^ > 0.9 for the compared groups demonstrated that these models were reliable and stable, thus providing an excellent explanation for the metabolic variations in the three cultivars, which could be used to further screen out the DAMs using VIP analysis.

### 3.3 Differential metabolite analysis of quinoa grains

**Fig. 3:**
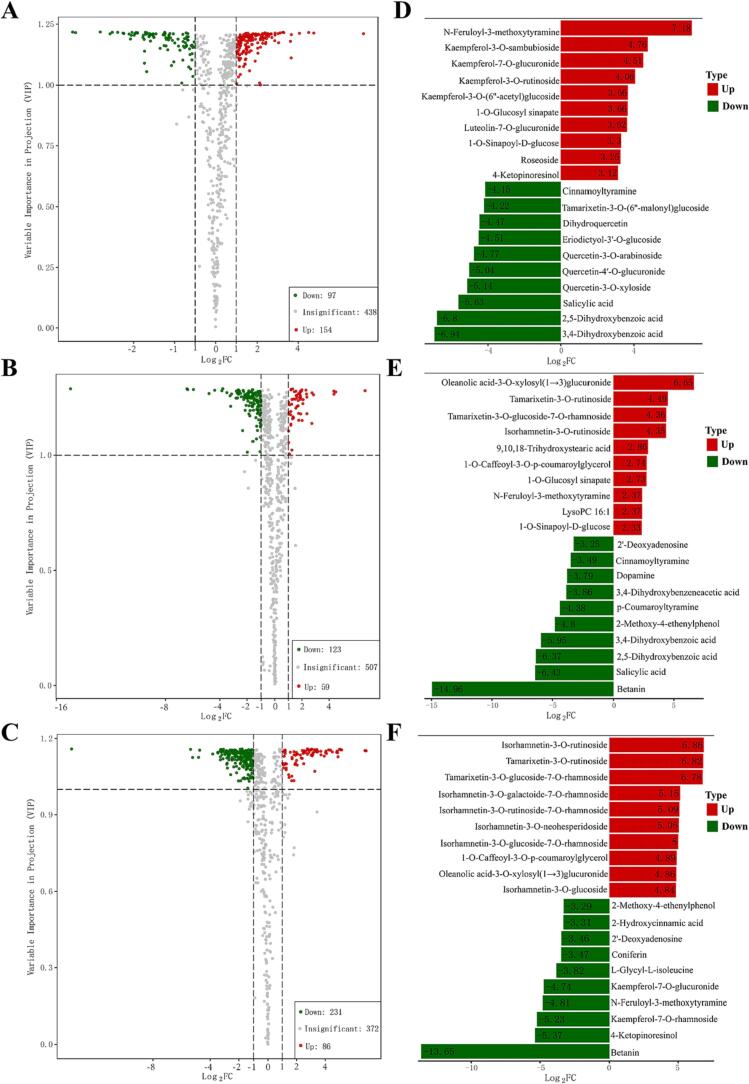
Chemical differences among the three differently colored quinoa grains. A, B, and C: The volcano plot shows the differential metabolite expression levels among the Black, White, and Red samples. Red, green, and gray dots indicate upregulated, downregulated, and insignificant differentially expressed metabolites, respectively. D, E, and F: Top 10 upregulated and downregulated compounds in Black and Red, Black and White, and Red and White, respectively. (For interpretation of the references to color in this figure legend, the reader is referred to the web version of this article.).

Analyses of multiple samples revealed different patterns of metabolite accumulation. To identify the most meaningful DAMs among the three quinoa cultivars, the 689 annotated metabolites were screened based on fold change and VIP scores (Table S2). Metabolites with a fold change of ≥2 or ≤0.5 and VIP ≥ 1 were defined as DAMs. There were 251 DAMs (154 upregulated, 97 downregulated) between Black and Red (Fig. 3A), 182 DAMs (59 upregulated, 123 downregulated) between Black and White (Fig. 3B), and 317 DAMs (86 upregulated, 231 downregulated) between Red and White (Fig. 3C). Next, DAMs from the three comparison groups (Black vs Red, Black vs White, and Red vs White) were classified into 11, 10, and 11 different categories, respectively. The DAMs in different cultivars of quinoa grains are shown in Table S2. Notably, most DAMs were flavonoids (29.10 %) and phenolic acids (16.30 %) when comparing Black and Red. Moreover, the comparative analysis revealed that amino acids and derivatives, alkaloids, organic acids, nucleotides and derivatives, phenolic acids, organic acids, and lignans and coumarins were significantly more abundant in black and red quinoa grains than in white grains.

However, the distribution of flavonoids and phenolic acids varied slightly among the three groups. Compared with white quinoa grains, most flavonoids (flavonols, flavones, dihydroflavones, and chalcones) in colored quinoa grains had lower levels, whereas several types of phenolic acids showed higher levels. Furthermore, the levels of alkaloids and lipids differed significantly among quinoa cultivars. For instance, the contents of lipids and most alkaloids (except diethanolamine, indole 3-acetic acid, and feruloylhistamine) were higher in red quinoa grains than those in black and white quinoa grains, respectively. Colored quinoa grains differed in their nutritional value and taste (Pereira et al., 2019). Therefore, we recommend selecting quinoa grains of different colors depending on specific bioactive compounds.

### 3.4 Changes in phenolic compounds in quinoa grains of different cultivars

Phenolics contribute significantly to the astringency, flavor, color, and oxidative stability of foods (Mutha, Tatiya & Surana, 2021). They are secondary plant metabolites that can be classified into free and bound forms (Cheynier et al., 2013). Free phenolics can be extracted into solution, whereas insoluble bound phenolics are covalently conjugated to structural components in the cell wall. In general, aqueous methanol is used to extract free phenolic compounds, whereas bound phenolics are efficiently collected via alkaline and acid hydrolysis (Tian, Nakamura, & Kayahara, 2004). Quinoa grains are known to contain a high concentration of phenolic chemicals and exhibit significant antibacterial, antioxidant, and anticancer activities, which are beneficial to human health (Pereira et al., 2020). Therefore, the composition and relative content of flavonoids and phenolic acids in different quinoa cultivars were investigated in this study.

Multivariate analyses such as HCA were employed to elucidate the differences in metabolic profiles of the three quinoa cultivars, which were determined using UPLC-QQQ-MS/MS in the positive/negative ionization mode. HCA revealed varying relative levels of flavonoids and phenolic acids in different cultivars in relation to phenotypic variation. The flavonoid heatmap is presented in Fig. S2A; flavonoids were classified into three main classes based on their accumulation patterns. The contents of flavonoids in class I, II, and III were highest in red, white, and black quinoa grains, respectively. Furthermore, phenolic acids could be classified into four main clusters based on HCA (Fig. S2B). Clusters I and II contained 56 phenolic acids, and the content of most metabolites in these clusters was highest in red quinoa grains. The levels of phenolic acids in cluster III were higher in the white cultivar than those in the black and red cultivar. Additionally, the black cultivar belonging to cluster IV showed the highest accumulation of phenolic acids.

Further, we studied the most significant flavonoid and phenolic acid changes in different quinoa cultivars. A comparison between Black and Red revealed a change factor in the range of 0.03–27.02 fold for 73 different flavonoids (25 upregulated and 48 downregulated). The top five upregulated flavonoids in red quinoa were kaempferol-3-*O*-sambubioside (27.02-fold), kaempferol-7-*O*-glucuronide (22.79-fold), kaempferol-3-*O*-rutinoside (16.68-fold), kaempferol-3-*O*-(6″-acetyl) glucoside (12.68-fold), and luteolin-7-*O*-glucuronide (12.28-fold). The top six downregulated flavonoids in red quinoa were tamarixetin-3-*O*-(6″-malonyl) glucoside (0.05-fold), dihydroquercetin (0.05-fold), eriodictyol-3′-*O*-glucoside (0.04-fold), quercetin-3-*O*-arabinoside (0.04-fold), quercetin-4′-*O*-glucuronide (0.03-fold), and quercetin-3-*O*-xyloside (0.03-fold). Most DAMs in Black vs Red were quercetin and kaempferol glycosides (Fig. 3D), indicating that black and red quinoa grains exhibit different pharmacological activities. Moreover, 28 differential flavonoids were identified between Black and White, with a fold change of 0.11–22.45. The top three flavonoids, namely tamarixetin-3-*O*-rutinoside (22.45-fold), tamarixetin-3-*O*-glucoside-7-*O*-rhamnoside (20.56-fold), and isorhamnetin-3-*O*-rutinoside (20.39-fold), were present at higher levels in white cultivars (Fig. 3E). Additionally, 69 differential flavonoids were detected between Red and White. As shown in Fig. 3F, the top 10 upregulated DAMs in white quinoa included eight flavonoids, with a fold change of 28.58–116.20. The top 10 downregulated DAMs in white quinoa included kaempferol-7-*O*-glucuronide (0.04) and kaempferol-7-*O*-rhamnoside (0.03). Consequently, our results suggested that the color of quinoa grains significantly affected on the flavonoid content. We can select and breed specific quinoa grain compositions by monitoring the metabolomic changes between different cultivars, as previously reported by Liu et al. (2021). Moreover, the antioxidant activity of quinoa is associated with phenolic compounds; the differences in flavonoid content among the three quinoa varieties could be attributed to their varying antioxidant activities (Piñuel et al., 2019).

There were 41 (23 upregulated, 18 downregulated) significantly different phenolic acids between Black and Red, 37 (11 upregulated, 26 downregulated) between Black and White, and 29 (4 upregulated, 25 downregulated) between Red and White. Most phenolic acids were found at higher levels in red quinoa than those in black and white quinoa. This finding is similar to that of a previous report that showed higher concentrations of phenolics in red quinoa than those in black and white quinoa (Liu et al., 2020). In this study, black and red grains had markedly higher contents of *p*-coumaric acid and ferulic acid. Four chlorogenic acid isomers, namely, neochlorogenic acid, cryptochlorogenic acid, isochlorogenic acid C, and isochlorogenic acid B, were identified in quinoa grains from different cultivars. Meanwhile, the levels of 5-*O*-*p*-coumaroylquinic, ferulic acid, vanillic acid, 4-hydroxybenzoic acid, and syringic acid in red quinoa grains were 1.03–9.07-fold higher than those in black and white grains. Further, the protocatechuic acid content in Black was 123.10-fold and 61.79-fold higher than those in Red and White, respectively. Therefore, we found a variation in the composition and content of aglycon phenolic acids among differently colored quinoa grains. Regarding glycosylated phenolic acids, the content of vanillic acid-4-*O*-glucoside in Red was 7.31-fold and 1.96-fold higher than that in Black and White, respectively. Likewise, the fold changes in *p*-coumaric acid-4-*O*-glucoside and ferulic acid-4-*O*-glucoside between red and white quinoa grains were >1.77.

In general, our study provided information on phenolic acid composition; we found that most phenolic acids in quinoa exist as conjugated forms, which is in agreement with the results of a previous research (Han et al., 2019). These results suggest that the content of glycosylated phenolic acids in quinoa grains contributes to their antioxidant properties. Additionally, 4-hydroxybenzoic acid and protocatechuic acid were identified as primary phenolic acids; the former was found at the highest level in red and white quinoa grains, whereas the latter had the highest level in black quinoa grains. Protocatechuic acid is a precursor in the synthesis of several complex substances, such as vanillin and anthocyanin 3-*O*-β-d-glucoside, and exhibits antibacterial, anti-inflammatory, and antioxidant activities (Shi, An, Jiang, Guan & Bao, 2006).

### 3.5 Crucial differential metabolites in quinoa grains of different colors

Quinoa grains were found to be rich in flavonoids and phenolic acids, and their contents were significantly affected by color genotypes (Pereira et al., 2020). Previous research identified betacyanins (betanin and isobetanin) as the pigments in red and black quinoa grains (Tang et al., 2015). In this study, there were 53 compounds were found to be the common differential metabolites between Black vs Red, Black vs White, and Red vs White (Fig. 5A), including 22 flavonoids, 5 phenolic acids, and 1 betacyanin (Table 1).

**Table 1:** Detailed information on the crucial differential metabolites identified and Pearson's correlation coefficients in the three differently colored quinoa grains.

Cultivars	NO.	Compounds	RT (Min)	Error (ppm)	Q1 (Da)	Q3 (Da)	Molecular Weight (Da)	Main fragments (Da)	Class	Betanin
*‘Yana’*										
Black	1	Betanin	2.08	0.91	551.2	389.1	550.143	389.1	Alkaloid	1
	2	p-Coumaraldehyde	4.25	−2.72	147.1	129.0	148.052	119.1, 129.0	Phenolic acid	0.609
	3	2-Methoxy-4-ethenylphenol	1.85	−3.92	151.1	91.1	150.068	91.1, 119.1	Phenolic acid	0.928**
	4	Eriodictyol	5.03	0.70	287.1	135.0	288.063	135.0, 151.0	Dihydroflavone	0.814**
	5	Eriodictyol-3′-O-glucoside	4.27	1.34	449.1	287.1	450.116	287.1, 151.0, 135.0	Dihydroflavone	0.689*
	6	Dihydroquercetin	4.23	−4.29	303.1	125.0	304.058	125.0, 285.0	Dihydroflavonol	0.736*
	7	Quercetin-3-O-xyloside	4.13	−0.92	435.1	303.1	434.085	303.1, 229.0	Flavonol	0.676*
	8	Quercetin-3-O-arabinoside	4.28	−1.39	433.1	300.0	434.085	151.0, 271.0, 300.0	Flavonol	0.642
	9	Quercetin-4′-O-glucuronide	3.78	0.84	477.1	301.0	478.075	151.0, 301.0	Flavonol	0.690*
	10	Diosmetin-7-O-rutinoside	4.15	0.82	609.2	301.0	608.174	129.1 301.1	Flavone	0.725*

*‘Pasan Ralle’*										
Red	11	Ferulic acid	4.03	−3.63	193.1	134.0	194.058	134.0, 149.1	Phenolic acid	0.140
	12	3-[(1-Carboxyvinyl) oxy] benzoic acid	3.92	1.45	207.0	119.1	208.037	119.1, 135.0	Phenolic acid	−0.395
	13	Kaempferol-7-O-rhamnoside	4.72	1.86	431.1	285.0	432.106	151.0, 285.0	Flavonol	0.104
	14	Kaempferol-3-O-(6″-acetyl) glucoside	4.37	1.02	489.1	285.0	490.111	285.0, 255.0	Flavonol	−0.153
	15	Kaempferol-3-O-sambubioside	3.96	−1.20	581.2	287.1	580.142	287.1, 449.1	Flavonol	−0.196
	16	Kaempferol-3-O-rutinoside	3.90	−0.84	593.2	285.0	594.158	285.0, 327.1	Flavonol	−0.197
	17	Apigenin-7-O-neohesperidoside	4.32	0.86	579.2	271.1	578.164	129.1, 271.1, 433.1	Flavone	−0.340

*‘QQ87’*										
White	18	1-O-Caffeoyl-3-O-p-coumaroylglycerol	5.35	−0.75	399.1	163.0	400.116	163.0, 253.1	Phenolic acid	−0.662
	19	Isorhamnetin-7-O-glucoside	4.21	−0.83	479.1	317.1	478.111	145.0, 317.1	Flavonol	−0.557
	20	Isorhamnetin-3-O-glucoside	4.23	−1.67	479.1	317.1	478.111	279.2, 317.1	Flavonol	−0.559
	21	Isorhamnetin-3-O-rutinoside	4.09	1.12	625.2	317.1	624.162	317.1, 479.1	Flavonol	−0.707*
	22	Isorhamnetin-3-O-neohesperidoside	3.54	−0.48	625.2	317.1	624.169	317.1, 479.1	Flavonol	−0.617
	23	Isorhamnetin-3-O-glucoside-7-O-rhamnoside	3.50	−2.08	625.2	317.1	624.169	145.0, 317.1, 463.1	Flavonol	−0.593
	24	Isorhamnetin-3-O-galactoide-7-O-rhamnoside	3.44	0.48	625.2	317.1	624.169	317.1, 463.1	Flavonol	−0.590
	25	Isorhamnetin-3-O-rutinoside-7-O-rhamnoside	3.70	−0.39	771.2	317.1	770.227	317.1, 479.1, 625.2	Flavonol	−0.549
	26	Tamarixetin-3-O-rutinoside	4.18	−0.96	623.2	315.1	624.168	300.0, 315.1	Flavonol	−0.709*
	27	Tamarixetin-3-O-glucoside-7-O-rhamnoside	4.01	0.80	625.2	317.1	624.169	317.1, 479.1	Flavonol	−0.678*
	28	Quercetin-7-O-rutinoside-4′-O-glucoside	3.49	0.26	773.2	303.1	772.206	303.1, 465.1	Flavonol	−0.544

Note: “*” and “**” mean p < 0.05 and p < 0.01, respectively.

**Fig. 4:**
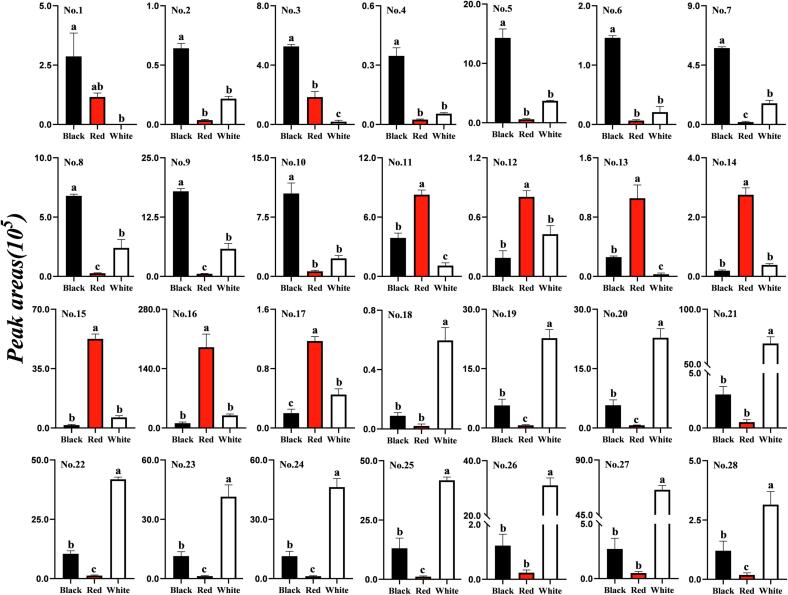
Peak areas of 28 different metabolites identified in three quinoa cultivars. Duncan's test was used to evaluate the significances. The compound number corresponds to the same in Table 1. Values with different letters indicate significant differences (values are expressed as mean ± SD, *P* < 0.05).

As shown in Fig. 4, betanin was detected only in black and red quinoa grains, and no anthocyanins were discovered in any samples. These results suggest that betanin acts as part of pigment composition in tested red and black quinoa cultivars, which is consistent with the results of the study by Tang et al (Tang et al., 2015). The concentrations of compounds 1–10 were significantly higher in black grains than those in red or white quinoa cultivars. In particular, the betanin level in the black quinoa cultivar was approximately 2.50 times higher than that in red samples, and the contents of quercetin glycoside derivatives were 3.08–35.37 times higher than those in red or white quinoa grains. An important difference noted in previous studies is that quercetin derivatives have beneficial biological functions, particularly antioxidant and anti-inflammatory activities (Tang et al., 2015). Meanwhile, the contents of compounds 11–17, including four kaempferol derivatives, were 2.12–37.45 times higher in red quinoa than those in black and white cultivars. A total of 11 metabolites (compounds 18–28) were present at a higher level in white quinoa grains than those in red and black quinoa grains. The levels of all isorhamnetin derivatives in white quinoa were 2.93–116.20 times higher than those in black and red grains.

**Fig. 5:**
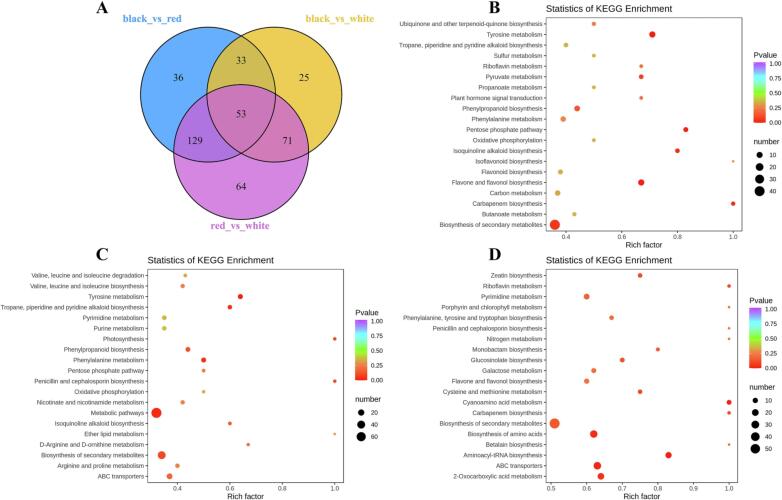
Venn diagram and pathway analysis of differentially accumulated metabolites in the three groups. A: The Venn diagram shows the overlapping and cultivar-specific differentially accumulated metabolites in the Black, White, and Red samples. B–D: KEGG pathway enrichment of the differentially accumulated metabolites among the groups (Black vs Red, Black vs White, and Red vs White). Each bubble in the plot represents a metabolic pathway whose abscissa and bubble size jointly indicate the magnitude of the impact factors of the pathway. A larger bubble size indicates a larger impact factor. The bubble colors represent the p-values of the enrichment analysis, with darker colors showing a higher degree of enrichment. (For interpretation of the references to color in this figure legend, the reader is referred to the web version of this article.).

Previous studies have identified that flavonoids and phenolic acids are important copigments of anthocyanin (Klisurova et al., 2019, Shiono et al., 2005, Tanaka et al., 2008), but the copigment effects of phenolic compounds on betacyanins were inadequately researched. To explore the relationship among betacyanins, flavonoids, and phenolic acids, Pearson’s correlation coefficients were calculated for the contents of these representative compounds (Table 1). As shown in Table 1, metabolites 1–10 exhibited positive correlations with betanin, seven of which displayed significant or extremely significant positive correlations (*P < 0.05* or *P < 0.01*). There were no significant correlations between the levels of betanin and compounds 11–17, which showed the highest levels in red quinoa grain. Meanwhile, the levels of compounds 18–28 were negatively correlated with betanin level, of which, two glycosylated tamarixetins and one glycosylated isorhamnetin showed significant negative correlations with betanin (*P < 0.05*). Altogether, our results showed that the contents of several flavonoids and phenolic acids were significantly and positively correlated with the betanin concentration. Transcriptomic and metabolomic analyses by Liu et al. (2021) found 18 flavone metabolites and 25 flavonoid-related genes as the main differential metabolites and genes in quinoa grains of four colors (black, red, yellow, and white). Combined with the fact that betacyanins are functionally similar to anthocyanins and can act as substitutes for plant color (Li, Meng, Zhu & Li, 2019), we conjecture that flavonoids and phenolic acids could act as copigments with betacyanins. However, this hypothesis requires further experimental verification.

### 3.6 Metabolic pathway analysis of differential metabolites in the grains of three quinoa cultivars

The KEGG database is an effective tool for analyzing metabolic pathways and the relationship among them to represent different metabolic pathways as diagrams (Wang et al., 2020). Accordingly, DAMs in the differently colored grain samples were subjected to enrichment analysis using KEGG spider to acquire comprehensive functional information. The DAMs for Black vs Red, Black vs White, and Red vs White were involved in 73, 65, and 81 pathways, respectively. The top 20 metabolic pathways for the three comparison groups were associated with metabolic pathways, biosynthesis of secondary metabolites, ABC transporters, biosynthesis of amino acids, flavone and flavonol biosynthesis, phenylpropanoid biosynthesis, and flavonoid biosynthesis (Fig. 5B–D; Table S3). The most common results were “metabolic pathways” (66/92, 71.74 %, Black vs Red; 75/90, 83.33 %, Black vs White; 103/144, 71.53 %, Red vs White), followed by “biosynthesis of secondary metabolites” (40/92, 43.48 %, Black vs Red; 38/90, 42.22 %, Black vs White; 57/144, 39.59 %, Red vs White), and “ABC transporters” (11/92, 11.96 %, Black vs Red; 15/90, 16.67 %, Black vs White; 26/144, 18.06 %, Red vs White). Previous reports have indicated that ABC transporters are related to the transport and accumulation of secondary plant metabolites, such as flavonoids, terpenoids and alkaloids (Yazaki, 2006). Furthermore, the involvement of structural genes in ABC transporters is well documented in maize (Marrs et al., 1995, Otani et al., 2005). The differential metabolites of flavonoids and phenolic acids were analyzed using the KEGG database to further characterize the interactions among the groups. The results of KEGG enrichment analysis were consistent with the “phenylpropanoid biosynthesis”, “isoflavonoid biosynthesis”, “flavonoid biosynthesis”, and “flavone and flavonol biosynthesis” (Fig. S3), which are in agreement with the results of a previous report (Liu et al., 2021).

## 3 Conclusions

In this study, the metabolic profiles of three quinoa cultivars with various grain colors were systematically evaluated to investigate the differences in metabolites based on the widely targeted metabolomics approach. A total of 689 metabolites in whole quinoa grains were detected and annotated, which included 126 lipids, 1 steroid, 75 organic acids, 21 terpenoids, 60 alkaloids, 3 tannins, 62 others, 7 lignans and 7 coumarins, 102 flavonoids, 57 nucleotides and derivatives, 97 phenolic acids, and 71 amino acids and derivatives.

Quinoa is a significant food because of its high nutritional value and gluten-free nature. However, the differences in the metabolites produced by differently colored quinoa cultivars have not been thoroughly researched. Our research focused on the composition and differences in metabolic profiles of differently colored quinoa grains, especially in terms of their flavonoids and phenolic acids contents, to determine the differences in their nutritional value. “Metabolic pathways”, “biosynthesis of secondary metabolites”, and “ABC transporters” were the significantly enriched pathways. Overall, this research not only advances our knowledge of the metabolic mechanisms in quinoa but also aids in assessing metabolic quality by laying a firm foundation for further cultivation of fine quinoa cultivars.

## Funding

This research was supported by the 10.13039/501100001809National Natural Science Foundation of China No: 32170279 and 31570246, Fundamental Research Funds for the Central Universities No: 2572019CT03, CACMS Innovation Fund (CI2021A04112) and the National Key Research and Development Project (2019YFE0108700, China).

## Supplementary material

Detailed information on the 689 metabolites identified in quinoa of different grain colors is listed in Table S1. Differentially accumulated metabolites between Black and Red, Black and White, and Red and White (Table S2). Detailed information on KEGG enrichment analysis from differentially accumulated metabolites in three quinoa cultivars (Table S3). Total ion current (TIC) of QC samples and Pearson’s correlation coefficient analysis of Black, Red, White, and mixed quality control samples (Fig. S1). Clustering heatmap of all flavonoids and phenolic acids (Fig. S2). KEGG annotations and enrichment of differential flavonoid and phenolic acid metabolites (Fig. S3).

## CRediT authorship contribution statement

**Guangtao Qian:** Conceptualization, Methodology, Formal analysis, Investigation, Writing – original draft. **Xiangyu Li:** Supervision, Investigation, Funding acquisition. **Heng Zhang:** Resources, Supervision. **Hailong Zhang:** Investigation, Validation. **Jingwen Zhou:** Investigation, Validation. **Xiaohui Ma:** Investigation, Validation. **Wei Sun:** Supervision. **Wei Yang:** Methodology. **Ruikun He:** Methodology. **Atia-tul Wahab:** Investigation, Validation. **Huihua Wan:** Supervision, Investigation, Writing – review & editing. **Lixin Li:** Conceptualization, Supervision, Project administration, Methodology, Writing – review & editing, Funding acquisition.

## Declaration of Competing Interest

The authors declare that they have no known competing financial interests or personal relationships that could have appeared to influence the work reported in this paper.

## Data Availability

No data was used for the research described in the article.
