# Supplementary material for: Metabolomics analysis reveals the accumulation patterns of flavonoids and phenolic acids in quinoa (Chenopodium quinoa Willd.) grains of different colors
Source: Food Chem X. 2023 Feb 6;17:100594. doi: 10.1016/j.fochx.2023.100594 (PMC9945449; doi:10.1016/j.fochx.2023.100594)
Supplement: Supplementary data 1 [file mmc1.docx]

**Metabonomics analysis reveals the accumulation patterns of flavonoids and phenolic acids in quinoa (*Chenopodium quinoa* Willd.) grains of different colors**

Guangtao Qian^a,1^, Xiangyu Li^b,1^, Hailong Zhang^a^, Jingwen Zhou^a^, Xiaohui Ma^a^, Wei Sun^c^, Wei Yang^c^, Ruikun He^f^, Heng zhang^d^, Atia-tul-Wahab^e^, Huihua Wan^c^*, Lixin Li^a^*

^1^ These authors contributed equally to this work.

^a^ Key Laboratory of Saline-alkali Vegetation Ecology Restoration, Ministry of Education, College of Life Sciences, Northeast Forestry University, Harbin 150040, China

^b^ Institute of Crop Resources, Heilongjiang Academy of Agricultural Sciences, Harbin 150086, China

^c^ Key Laboratory of Beijing for Identification and Safety Evaluation of Chinese Medicine, Institute of Chinese Materia Medica, China Academy of Chinese Medical Sciences, Beijing 100700, China

^d^ State Key Laboratory of Plant Molecular Genetics, Shanghai Center for Plant Stress Biology, Center for Excellence in Molecular Plant Sciences, Chinese Academy of Sciences, Shanghai 201602, China

^e^ Center for Molecular Medicine and Drug Research, International Center for Chemical and Biological Sciences, University of Karachi, Karachi 75270, Pakistan

^e^ Byhealth Institute of Nutrition & Health, Guangzhou 510663, China

***Corresponding author:**

Prof. Huihua Wan

Institute of Chinese Materia Medica, China Academy of Chinese Medical Sciences

No.16 Nanxiaojie, Dongzhimen Nei Ave, Dongcheng District, Beijing, 100700, China

Tel: +86 15201196960; E-mail: [hhwan@icmm.ac.cn](mailto:hhwan@icmm.ac.cn)

Prof. Lixin Li

Key Laboratory of Saline-alkali Vegetation Ecology Restoration, Ministry of Education, College of Life Sciences, Northeast Forestry University

No.26 Hexing Road, Xiangfang District, Harbin 150040, China

Tel: +86 15546308809; E-mail: lixinli0515@nefu.edu.cn


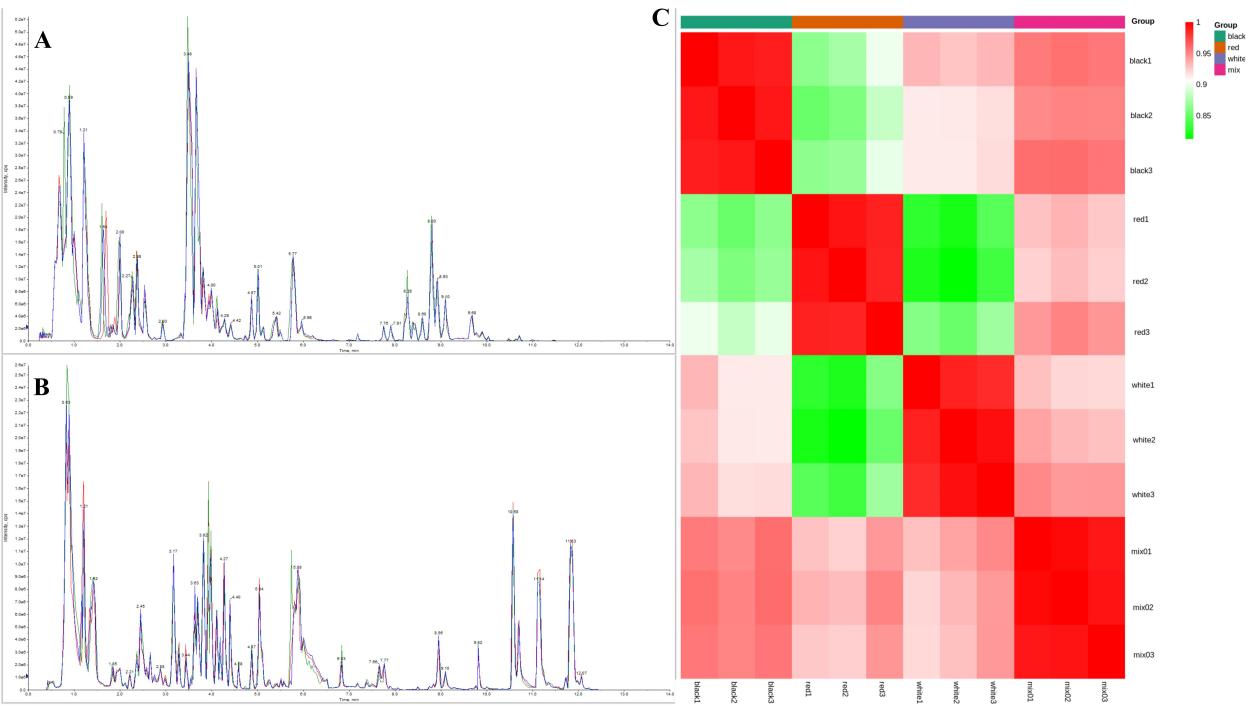


**Figure S1.** The total ion current (TIC) of the QC samples in positive ion mode (A) and negative ion mode (B), and Pearson's correlation coefficient analysis of Black, Red, White, and Mix quality control sample (C).


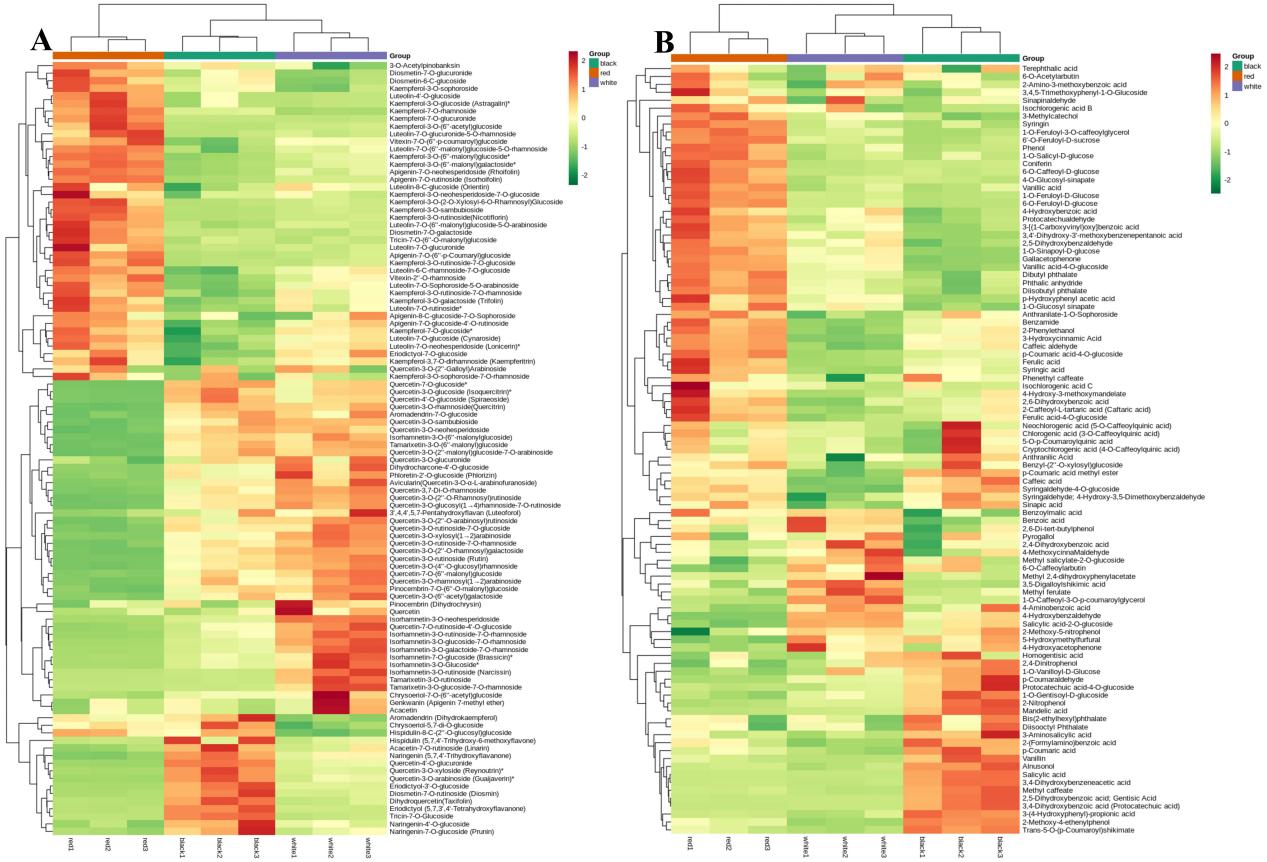


**Figure S2.** Clustering heat map of all flavonoids (A) and phenolic acids (B). The values of metabolites were normalized and shown as a color scale. The high and low metabolite levels were represented as red and green scales.


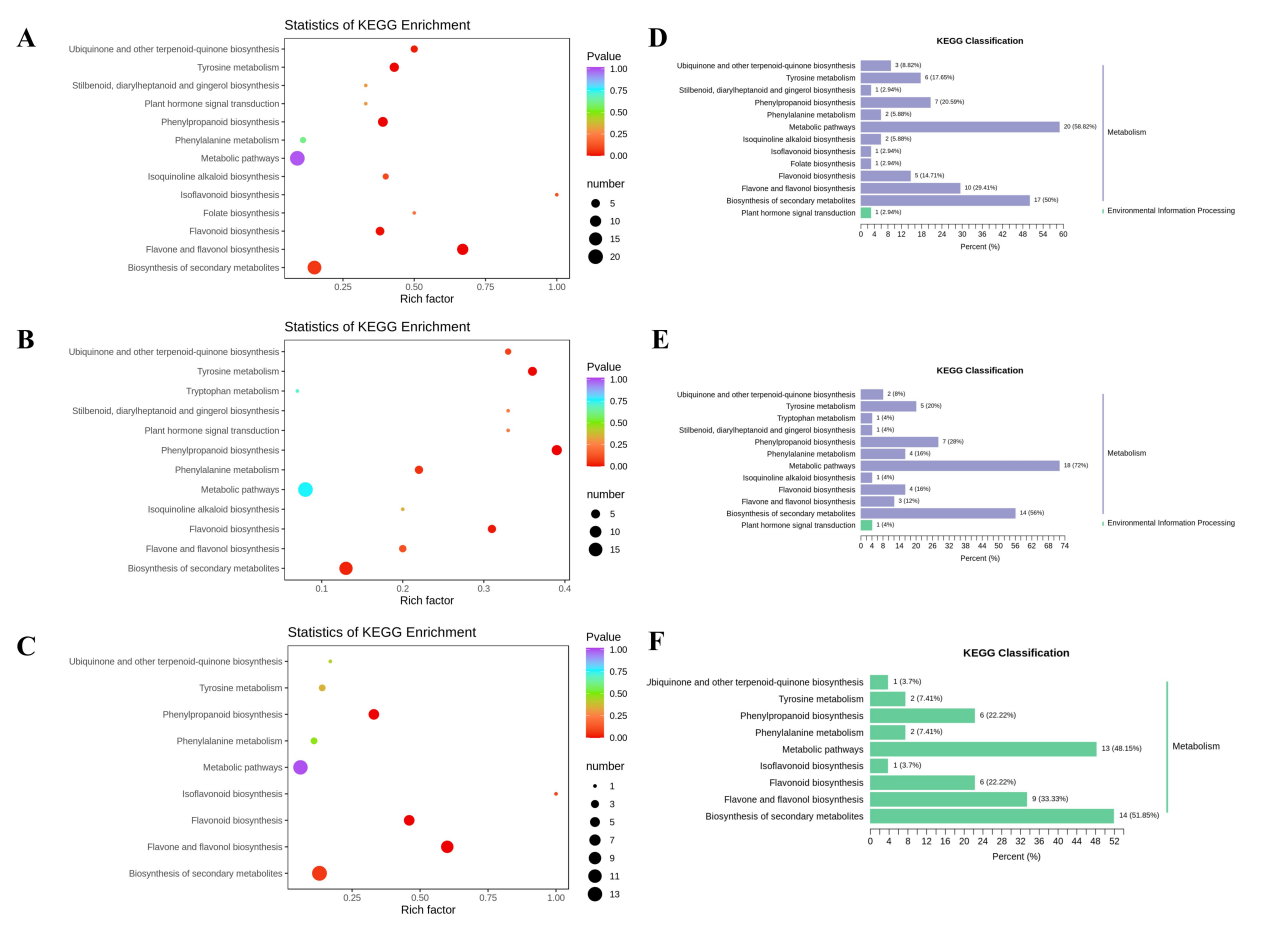


**Figure S3.** KEGG annotations and enrichment of differential flavonoid and phenolic acid metabolites. A-C: KEGG pathways enrichment of differential flavonoid and phenolic acid metabolites between the comparison groups ( Black vs. Red, Black vs. White, and Red vs. White). D-F: The differential flavonoid and phenolic acid metabolites KEGG classification of the comparison group Black vs. Red, Black vs. White, Red vs. White, respectively.
